# Olfactory Receptor OR2K2 Expression in Human Choroid Plexus as a Potential Marker in Early Sporadic Alzheimer’s Disease

**DOI:** 10.3390/genes15030385

**Published:** 2024-03-21

**Authors:** Victoria Cunha Alves, Joana Figueiro-Silva, Ramon Trullas, Isidre Ferrer, Eva Carro

**Affiliations:** 1 Neurodegenerative Diseases Group, Hospital Universitario 12 de Octubre Research Institute (imas12), 28041 Madrid, Spain; 2 Network Center for Biomedical Research, Neurodegenerative Diseases (CIBERNED), 28029 Madrid, Spain; 3 Institute of Medical Genetics, University of Zurich, 8952 Zurich, Switzerland; jfigueiros@gmail.com; 4 Department of Molecular Life Science, University of Zurich, 8952 Zurich, Switzerland; 5 Department of Cell Death and Proliferation, Institut d’Investigacions Biomèdiques de Barcelona, Consejo Superior de Investigaciones Científicas (CSIC), Institut d’Investigacions Biomèdiques August Pi I Sunyer (IDIBAPS), 08036 Barcelona, Spain; 6 Institute of Neuropathology, Bellvitge University Hospital-IDIBELL, 08908 Barcelona, Spain; 7 Department of Pathology and Experimental Therapeutics, University of Barcelona, 08007 Barcelona, Spain; 8 Neurobiology of Alzheimer’s Disease Unit, Functional Unit for Research into Chronic Diseases, Instituto de Salud Carlos III, 28222 Madrid, Spain

**Keywords:** olfactory receptor, OR2K2, transcriptomic, autophagy, choroid plexus, Alzheimer’s disease

## Abstract

Epithelial cells comprising the choroid plexus (CP) form a crucial barrier between the blood and the cerebrospinal fluid, thereby assuming a central position in brain homeostasis and signaling. Mounting evidence suggests that the impairment of CP function may be a significant contributor to Alzheimer’s disease (AD) pathogenesis. CP function relies on the expression of specific receptors, and the potential involvement of olfactory receptors (ORs) and taste receptors (TASRs) in chemical surveillance within the CP is being investigated. Previous studies have implicated ORs and TASRs in neurodegenerative disorders like AD, although the direct evidence of their expression in the human CP remains to be established. In this study, we conducted a transcriptomic analysis encompassing eleven *ORs* and *TASRs* in the CP, comparing samples from healthy age-matched controls to those from patients with AD spanning Braak stages I to VI. Among these receptors, a striking finding emerged—*OR2K2* exhibited robust expression, with a statistically significant upregulation noted at Braak stage I. Surprisingly, at the protein level, OR2K2 showed a significant decrease in both Braak stage I and VI. Additionally, we identified CP epithelial cells as the source of OR2K2 expression, where it colocalized with autophagy markers LC3 and p62. We postulate that OR2K2 could be subjected to degradation by autophagy in the early stages of AD, triggering a compensatory mechanism that leads to increased *OR2K2* mRNA transcription. This study uncovers a potential role for OR2K2 in AD pathogenesis, offering a novel perspective on the intricate dynamics at play in this neurodegenerative disorder.

## 1. Introduction

The choroid plexus (CP) is an intricate, secretory tissue nestled within the lumen of cerebral ventricles, distinguished by its complex composition—an interplay of a vascular stroma enveloped by a retinue of epithelial cells. While its primary role centers on the production of cerebrospinal fluid (CSF) [1], an emerging function of the CP lies in its remarkable ability to sense changes in CSF composition, a feature primarily attributed to the expression of specific receptors, enabling the CP to adapt dynamically through metabolic and transcriptomic adjustments [2]. Notable among these discerning receptors are olfactory receptors (ORs), vomeronasal receptors, and taste receptors (TASRs) [3].

ORs belong to the family of transmembrane domain G protein-coupled receptors, playing a pivotal role in detecting odorants and shaping our sense of smell. While their expression is primarily confined to the nasal cavity, their influence extends broadly across a wide spectrum of biological processes [4]. In the human brain, OR expression has been detected in select regions such as the frontal cortex, entorhinal cortex, and cerebellum, with their dysregulation linked to several neurodegenerative diseases, including Parkinson’s disease [5], Alzheimer’s disease (AD) [6], Progressive Supranuclear Palsy, and Creutzfeldt–Jakob disease [7]. However, in the human CP, evidence for OR expression, regulation, and function is currently lacking. Current knowledge arises from studies conducted on the CP of other species, as exemplified by *Olfr78*, the orthologue of human *OR51E2*, which exhibits high expression in macrophages of the mouse CP [8]. Additionally, in the rat CP, both mRNA and protein expressions of olfactory receptors and key components of their signaling pathway, including the olfactory G-protein, adenylate cyclase 3, and cyclic nucleotide-gated channel have been elucidated by Gonçalves et al. [9].

It becomes progressively evident that CP dysfunction may constitute a major contributing factor to AD. AD manifests as a progressive decline in cognitive abilities, accompanied by brain atrophy, the accumulation of amyloid β (Aβ) plaques in the brain’s parenchyma, and the formation of hyperphosphorylated tau protein aggregates within neurons [10]. Recent investigations, encompassing in vitro, in vivo, and human studies, have brought to light a spectrum of morphological modifications and inflammatory responses within the CP, among a myriad of other alterations. Additionally, the choroid plexus–CSF system has been shown to promote the clearance of toxic Aβ species from the brain, channeling them into CSF through specific transporters, thereby thwarting Aβ plaque formation [11,12,13]. 

Additionally, the investigation into the normal expression and functional dynamics of receptors such as OR and TASR within the CP of AD, including their potential involvement in the clearance of toxic Aβ species, remains largely unexplored.

In light of these revelations, our study seeks to delve into the potential expression and regulation of ORs and TASRs in their role as vigilant sentinels engaged in chemical surveillance within the CP and to decipher their modulation throughout the progression of AD. To achieve this, we conducted a transcriptomic analysis spanning the full spectrum of AD pathogenesis, from Braak stage I through to VI, thereby enabling us to track early and incipient alterations occurring during the progression of this complex disease.

## 2. Materials and Methods

**Tissue samples**

Human post-mortem CP tissue from 11 age-matched healthy controls and 26 donors diagnosed with AD were provided by the Institute of Neuropathology Brain Bank (HUB-ICO-IDIBELL Biobank, Barcelona, Spain), the Netherlands Brain Bank (NBB, Amsterdam, The Netherlands), and Banco de Tejidos, Fundación CIEN (Centro de Investigación de Enfermedades Neurológicas, Madrid, Spain). All samples were acquired following the relevant guidelines and regulations and approved by the research ethics committee of each responsible institution, including the Research Ethics Committee of Hospital Universitario 12 de Octubre (18/459). The neuropathological diagnosis of sporadic AD was based on neurofibrillary tangle pathology and Aβ plaques [14]. Age-matched healthy controls displayed no signs of neurological pathology. Demographic and clinical data are summarized in Table 1.

***APOE* genotype**

Genomic DNA was extracted with DNeasy Blood and Tissue Kit (Qiagen, Hilden, Germany) according to the manufacturer’s instructions. Identification of *APOE* ϵ2, ϵ3, and ϵ4 alleles was carried out by Taqman assays using the LightMix ApoE C112R and R158C Kit (Roche Diagnostics, Berlin, Germany) on a LightCycler 480 II Instrument (Roche, Mannheim, Germany).

**Selfie-digital PCR**

The absolute number of mRNA transcripts per gene was measured by Selfie-digital PCR according to Podlesniy and Trullas [15], with slight modifications. Briefly, CP tissue samples were solubilized with DireCtQuant 100ST (DireCtQuant, Lleida, Spain) at a 1:100 ratio [tissue weight (g)/extraction reagent volume (ml)]. Sample lysates and gene-specific primers complementary to RNA transcripts were pre-annealed in duplicate, and strand-specific reverse transcription was performed with (RT^+^) and without (RT^−^) reverse transcriptase. DNA was digested with FastDigest AluI or FastDigest SaqAI restriction enzymes (Thermo Fisher Scientific, Waltham, MA, USA) depending on the targeted sequence, and the reactions were partitioned and emulsified for droplet generation. Finally, the absolute number of transcripts in relationship with their self-encoding gene was calculated based on the ratio of positive (containing the amplified target) to total droplets. Non-template controls were included in all steps of the procedure. Primer sequences are listed in Table S1.

**Western blotting**

CP tissue was homogenized in RIPA lysis buffer [50 mM Tris-HCl, pH 8.0; 150 mM NaCl; 1% NP-40; 0.5% sodium deoxycholate; 0.1% SDS; protease and phosphatase inhibitors cocktail (Roche, Basel, Switzerland)], and total protein concentration was determined using the Pierce BCA Protein Assay Kit (Thermo Fisher Scientific, Waltham, MA, USA). A 20–25 µg quantity of protein was reduced in 100 mM DTT in Laemmli buffer (1% LDS, Bio-Rad, Hercules, CA, USA) for 30 min at 25 °C and resolved on 12% SDS-PAGE gels and transferred to PVDF membranes. After blocking, membranes were incubated with primary antibodies overnight at 4 °C and with the appropriate horseradish peroxidase (HRP)-conjugated secondary antibodies for 1 h at room temperature. Chemiluminescence signal from immunocomplexes was generated using Clarity Western ECL substrate (Bio-Rad, Hercules, CA, USA). The antibodies used were as follows: rabbit anti-OR2K2 (C-terminal, ABIN3186065, antibodies-online GmbH, Aachen, Germany), 1:1000; rabbit anti-OR2K2 (N-terminal, OAAJ05957, Aviva Systems Biology, San Diego, CA, USA), 1:2000; HRP-conjugated mouse anti-β actin (ab49900, Abcam, Cambridge, UK) 1:25,000; and HRP-conjugated goat anti-rabbit (G21234, Thermo Fisher Scientific, Waltham, MA, USA), 1:5000.

**Immunohistochemistry**

CP tissue was fixed for 24 h in 4% paraformaldehyde by immersion and was paraffin-embedded, and its free-floating 4 µm thick sections were obtained using a microtome (Leica). Heat-induced epitope retrieval was performed at 100 °C for 15 min in 0.2 M citrate. All primary antibodies were diluted in 0.1 M phosphate buffer containing 0.5% bovine serum albumin and 0.5% Triton X-100. The following primary antibodies were used at 1:500 dilution: rabbit anti-OR2K2 (DF5075, Affinity Biosciences, Cincinnati, OH, USA), mouse anti-p62/SQSTM1 (D5L7G, Cell Signaling Technology, Brookline, MA, USA), and mouse anti-LC3B (E5Q2K, Cell Signaling Technology, Brookline, MA, USA). After overnight incubation, primary antibody staining was revealed using fluorescence-conjugated secondary antibodies from Life Technologies (Carlsbad, CA, USA): Alexa Donkey anti-mouse 488 (A21202) and Alexa Goat anti-rabbit 555 (A27039). Finally, the slices were mounted with Immunoselect Antifading Mounting Medium with DAPI (SCR-038448, BioTrend, Köln, Germany). For the negative control, sections were treated following the previously outlined process, except for omitting the primary antibody. Fluorescent images were acquired with a Stellaris Laser Scanning confocal microscope, using the HCPLAPO 63X 1.4 NA objective, employing a single scan, with the pinhole size set at AIRY1/95.5 µm. For the acquisition of fluorescence images, a fixed exposure was maintained for all samples in all the experiments.

**Statistical analysis**

Statistical analyses were performed using GraphPad Prism, version 6.0 (GraphPad Software, La Jolla, CA, USA), and IBM SPSS Statistics for Windows, version 20 (IBM Corp., Armonk, NY, USA). Data are presented as mean values ± standard error of the mean (SEM). *p* < 0.05 was considered statistically significant. Applied statistical tests for each experiment are mentioned in the corresponding figure legends.

## 3. Results

### 3.1. OR and TAS2R Gene Expression in CP of Control and AD

Building upon our prior investigations, wherein we disclosed differential expressions of *ORs* and *TAS2Rs* in the orbitofrontal cortex of individuals with AD [6], along with the discovery of *OR7A5* expression in the middle frontal gyrus [16], our focus shifted to the CP, where we examined eleven distinct genes: olfactory receptors (*OR2K2*, *OR2H2*, *OR1L8*, *OR13A1*, *OR7A17*, and *OR7A5)*, bitter taste receptors (*TAS2R14* and *TAS2R5)*, and type 1 taste receptors (*TAS1R1*, *TAS1R2*, and *TAS1R3*). *OR* genes were selected based on gene families and chromosomal distribution, whereas *TAS2R* genes were selected based on the number of known ligands. A detailed explanation of the selection rationale can be found in Alves et al. [6]. Selfie-dPCR enables the detection of absolute transcript numbers, rendering it particularly advantageous for genes with low expression levels, such as *OR* and *TASR*. Moreover, it obviates the necessity for employing housekeeping genes, as stipulated by the MiQE guidelines for qPCR experiments [17]. 

**Figure 1:**
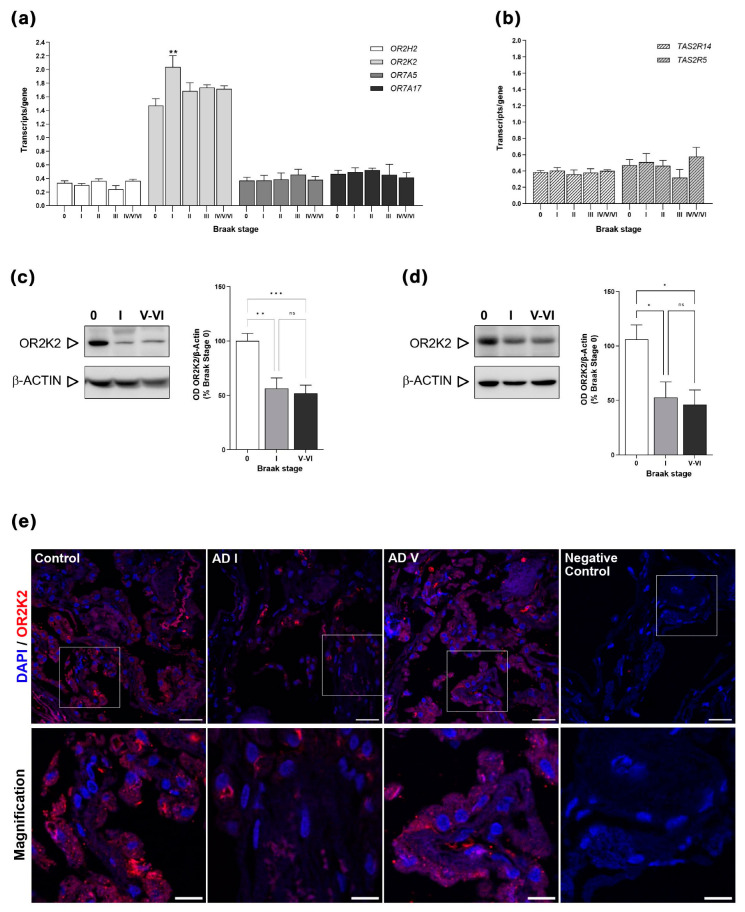
OR2K2 expression in CP. (**a**,**b**) Absolute measurement of *OR* (**a**) and *TASR* (**b**) gene transcription with selfie-dPCR. (**c**–**e**) OR2K2 relative protein expression in choroid plexus. Representative Western blot and relative quantification using C-terminal (**c**) and N-terminal (**d**) OR2K2 antibodies. Full immunoblots are presented in Figure S2. * *p* < 0.05; ** *p* < 0.01; *** *p* < 0.001, One-way ANOVA followed by Bonferroni’s multiple comparison test. Ns, not significant. (**e**) Representative confocal microscopic images of choroid plexus epithelial cells demonstrating the expressions of OR2K2 (red) and nuclear stain DAPI (blue) in control subject (80-year-old male) and patients with AD (76-year-old male, AD Braak I, and 82-year-old male, AD Braak V). For the negative control, the choroid plexus section was incubated without primary antibody. Scale bar, 100 μm. In the lower panel, a magnification of the region is indicated in the white boxes in the upper panel. Scale bar, 20 μm. AD, Alzheimer’s disease.

Our exploration revealed that several olfactory receptor genes, specifically *OR2H2*, *OR2K2*, *OR7A5*, and *OR7A17*, as well as bitter taste receptors genes *TAS2R5* and *TAS2R14*, are expressed within the CP milieu. Remarkably, the overall gene expression of both *OR* (Figure 1a) and *TAS2R* (Figure 1b) remained at a subdued level, with fewer than 0.6 transcripts per gene detected. The notable exception was *OR2K2*, which exhibited a more robust expression, with 1–2 transcripts per gene. Our investigations unveiled an absence of sexual dimorphism in the expression of the selected genes (Figure S1). No detection was observed for *OR1L8*, *OR13A1*, and type 1 taste receptors (*TAS1R1*, *TAS1R2*, and *TAS1R3*). Furthermore, for those genes exhibiting low expression levels, no Braak-stage-associated regulation was discerned. Notably, *OR2K2* stood out as the exception, exhibiting a statistically significant upregulation at Braak stage I (*p* < 0.01), only to revert to control levels as we advanced through subsequent Braak stages (Figure 1a). Notably, attempting to quantify gene expression through qPCR for the eleven analyzed *OR* and *TASR* genes yielded either no detection or it was outside the detection limit due to amplification at late cycles. Consequently, *OR* and *TASR* gene expression and subtle differences in *OR2K2* expression appear to be exclusively detectable with dPCR.

Furthermore, we examined the correlation between *OR2K2* expression and the *APOE* genotype. Spearman’s rho correlations showed no statistically significant associations between the gene expression and either *APOE* genotype or carrying an *APOE4* allele.

### 3.2. OR2K2 Protein Is Reduced in the Choroid Plexus at Early Stages of AD

Subsequently, by delving into *OR2K2* gene regulation with Braak stages, we explored OR2K2 protein levels. Employing Western blot analysis, our investigation unveiled a significant reduction in OR2K2 levels, both in early (*p* < 0.01) and advanced (*p* < 0.001) Braak stages in AD when compared to healthy controls (Figure 1c). We employed an additional antibody, bearing an N-terminal epitope. This approach yielded parallel results, affirming a significant reduction in OR2K2 levels at both Braak stages (*p* < 0.05, Figure 1d). The use of two antibodies stems from the dual transcripts of the *OR2K2* gene, wherein one transcript lacks the initial 30 amino acids compared to the other. The C-terminal antibody captures both transcripts, while the N-terminal antibody targets the longer one. Despite an anticipated 35 kDa band, the C-terminal antibody in Figure S2a reveals a robust 75 kDa signal, indicative of potential dimer formation. This aligns with documented tendencies of olfactory receptors, part of the G protein-coupled receptor superfamily, to exist as self-associated dimers or higher-order oligomers [18,19,20]. The disulfide bond region within OR2K2 and potential N-linked glycosylation offer explanations for the observed size discrepancies. The N-terminal antibody (Figure S2c) identifies a distinct 35 kDa band.

Further exploration employing immunofluorescence revealed OR2K2 expression in CP epithelial cells in healthy controls. In CP of AD cases (Braak stages I and V), a weak OR2K2 signal was discernable; therefore, we can only ascertain signal presence or absence from the displayed images and determine its localization (Figure 1e).

**Figure 2:**
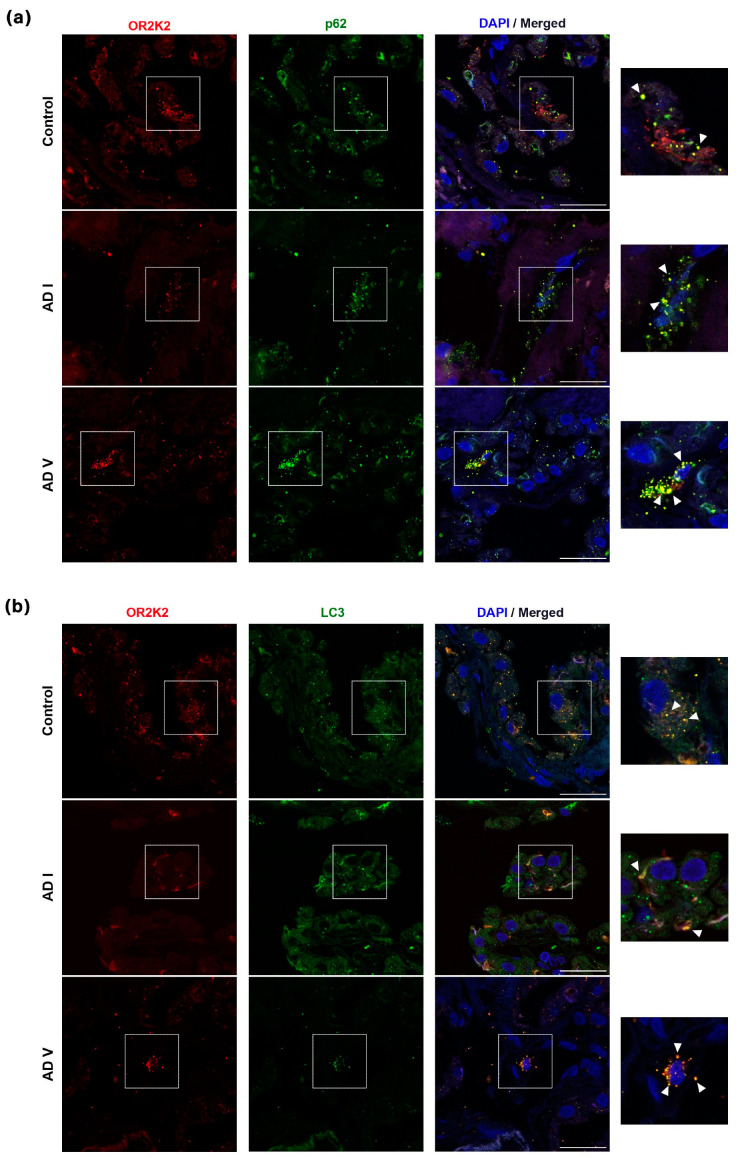
Colocalization of autophagic markers p62 and LC3 with OR2K2 in human CP. Immunohistochemistry of OR2K2 and p62 (**a**) and LC3 (**b**) was performed in CP sections. Representative confocal images of early (76-year-old male, AD Braak I) and advanced AD (82-year-old male, AD Braak V) cases compared with those from healthy donors (80-year-old male) are shown. Magnification insets from punctated boxes are presented on the right, with white arrows highlighting colocalization signals. OR2K2 (red), p62 (green), LC3 (green), and nuclear stain DAPI (blue). AD, Alzheimer’s disease. Scale bar, 100 μm.

Upon these observations, we then inquired whether OR2K2 was subject to degradation via autophagy. Immunofluorescence revealed the colocalization of OR2K2 with two autophagy markers, namely, p62 (Figure 2a) and LC3 (Figure 2b), within CP epithelial cells. In control subjects, this colocalization manifested in punctate-like structures, but the amount of colocalized OR2K2 with autophagy markers was more apparent in CP tissue from early AD (Braak stage I) and advanced AD (Braak stage V). Notably, this manifestation was marked by the presence of large autophagic vesicles, primarily in the advanced stages of AD. 

Taken together, the colocalization of OR2K2 with p62 and LC3 suggests that autophagy may contribute to the diminished levels of OR2K2. This observation aligns with findings from previous studies suggesting olfactory receptors are degraded by autophagy [21]. Moreover, increased *OR2K2* mRNA expression may be construed as a compensatory mechanism.

## 4. Discussion

To date, the landscape of transcriptomic analysis in human CP remains relatively uncharted. Earlier studies, based on microarray data from healthy human CP (GSE49974) [22], and a subsequent investigation including Braak stage VI donors (GSE61196) [23], did reveal a significant upregulation of *OR2H1* in the latter group, as assessed using the GEO2R feature of the GEO database. However, it is noteworthy that despite the coverage of *OR2H2*, *OR7A5*, and *OR7A17* in the array, *OR2K2* was absent from the analysis. Intriguingly, *OR13D1*, an important paralog of *OR2K2*, was also covered but failed to exhibit differential expression. 

In a more recent gene expression study encompassing healthy controls, advanced AD (Braak stage III–VI), frontotemporal dementia, and Huntington’s disease (GSE110226), *OR2K2* was indeed found to be expressed in the CP. However, no differential expression was discerned in AD versus healthy controls [24]. This finding aligns with our study, indicating the absence of *OR2K2* regulation from Braak stage III to VI in comparison to healthy controls. Notably, our study contributes a novel dimension by being the first to report the regulation of *OR2K2* expression in CP at the early stage of AD (Braak I). This insight enhances our understanding of the intricate landscape of CP transcriptomics within the context of early AD stages. Nevertheless, considering the diverse cellular composition of the CP, including epithelial, vascular, and mesenchymal cells, caution is warranted when comparing transcriptomic studies derived from bulk tissue.

Our hypothesis posits that the upregulation of *OR2K2* may serve as a compensatory mechanism in response to its protein targeting for degradation, as indicated by colocalization with autophagy markers LC3 and p62. A growing body of evidence has underscored the role of autophagy, a conserved mechanism for the degradation of numerous intracellular aggregates and proteins, in contributing to the pathogenesis of AD [25]. Notably, in CA1 hippocampal neurons from patients with AD, genes related to autophagosomes and lysosomes were found to be upregulated in early AD stages [26], suggesting that enhanced autophagy at this juncture may serve a protective role in response to stress. However, as AD progresses, autophagic flux becomes compromised [27]. In support of these findings, previous reports have documented elevated levels of LC3-II in cortical mid-frontal gray matter tissue [28] and prefrontal cortical tissue [29], with gradual increases observed from early-to-mild/moderate stages of AD when compared to neuropathologically normal age-matched controls. This trend is further underscored by observations of increased LC3-II and p62 in the brains of patients with *APOEε4* heterozygote [30] and patients with AD [31]. Moreover, in previous research conducted by our laboratory, significantly increased levels of LC3-II and p62 were observed in the CP across all stages of AD, suggesting increased autophagy induction [32]. 

Drawing upon prior research elucidating the phenomenon of OR being retained in the endoplasmic reticulum and subsequently undergoing degradation via the process of autophagy [21], we posit that the discernible reduction in OR2K2 expression throughout various stages of AD could plausibly stem from its degradation mediated by the autophagic pathway. Here, we find that the autophagy markers p62 and LC3 colocalize with OR2K2. However, other mechanisms (including the ubiquitin–proteasome system) may be involved in the degradation of these receptors.

Regarding the function of OR2K2 within the CP, it is worth noting that this receptor remains orphaned, with no ligand identified to date [33]. Furthermore, the process of signal transduction through ORs is notably reliant on several factors, including the particular OR involved, the specific cell type in which it operates, and the array of signaling components engaged, all of which convey different biological functions associated with these receptors [34]. Notable studies in this domain have suggested that certain ORs, such as OR2AT4 and OR2J3 in cultured lung epithelial cells, can mitigate inflammation by increasing the production of proinflammatory cytokines [35]. Similarly, OR4M1 in mouse primary cortico-hippocampal neurons has been shown to interfere with a key pathogenic mechanism in AD, specifically the phosphorylation of abnormal microtubule-associated tau protein, through a cAMP-dependent pathway and decreased c-Jun NH2-terminal kinase activity [36]. Although these investigations offer intriguing insights, it is important to note that while we may speculate about the potential role of OR2K2 in CP epithelial cells in regulating critical AD processes like inflammation and tau hyperphosphorylation, there is currently no empirical data directly substantiating this hypothesis. Consequently, the precise role of OR2K2 in human CP epithelial cells remains unknown, necessitating further in-depth inquiries to elucidate its influence, starting with examinations in healthy tissue and subsequently exploring its involvement in the early stages of the neurodegenerative process. 

This study offers valuable insights into the potential association between OR2K2 expression in CP and AD; however, it is crucial to acknowledge and address certain limitations that bear significance when interpreting the results and their implications. Firstly, it is imperative to highlight the modest sample size of patients with AD included in this study, particularly for the analysis of protein expression and the exploration of its potential degradation mechanism via autophagy. The approach taken in this regard involved a profiling analysis that can be characterized as more qualitative than quantitative. This brief report aims to outline the receptor and its regulation, laying the foundation for future in-depth studies. Subsequent investigations can delve into determining whether autophagy is the primary regulatory mechanism for this receptor or a part of a larger network of mechanisms. Furthermore, dissecting these mechanisms will shed light on the potential role of OR2K2 in AD pathogenesis.

Moreover, it is essential to consider the possibility that the decline in OR2K2 protein levels may be attributed to a range of post-transcriptional regulatory mechanisms. These mechanisms may include the potential involvement of microRNAs, translation-inhibitory proteins, ribosome sequestration, mRNA structural modifications, chemical modifications such as N6 adenosine methylation (m6A), and alternative mRNA splicing.

To solidify and expand upon these findings, further research is warranted. Additionally, a deeper comprehension of the role played by OR2K2 and other receptors in the blood–cerebrospinal fluid barrier and the surveillance of cerebrospinal fluid components in AD is necessary. This study sheds light on novel pathological changes in OR2K2 expression in early AD stages. While its immediate clinical implications are unclear, the findings provide pivotal groundwork for future research on OR2K2 as a potential diagnostic and therapeutic marker.

## Figures and Tables

**Table 1:** Demographic and clinical data of choroid plexus specimens (n = 37).

Variable	Braak 0	Braak I	Braak II	Braak III	Braak IV–VI	*p*-Value
Number of samples	11	7	7	3	9	
Sex male/female, n	6/5	4/3	3/4	3/0	4/5	
Age at death, mean years (range)	77.36 (70–85)	75.86 (70–82)	76.43 (72–80)	80.00 (77–82)	78.67 (73–83)	ns
PMI, mean hours ± SEM	7.18 ± 0.81	8.91 ± 2.20	7.00 ± 1.23	8.56 ± 2.04	5.35 ± 0.64	ns
*APOE4* carriers, n (%)	3 (27.3%)	0 (0%)	1 (7.1%)	1 (25%)	6 (75%)	

PMI, postmortem interval; SEM, standard error of the mean; ns, not significant. Braak 0 denotes absence of neurofibrillary pathology. Differences between groups were compared by the Bonferroni multiple comparison post hoc tests.

## Data Availability

All relevant data supporting the key findings of this study are available within the article and its Supplementary Information files or from corresponding authors on request.

## Supplementary Materials

The following supporting information can be downloaded at: https://www.mdpi.com/article/10.3390/genes15030385/s1, Figure S1: Comparison of chemoreceptors expression between males and females; Figure S2: Quantification of OR2K2 in the choroid plexus across Braak stages; Table S1: List of primer sequences used for selfie-digital PCR analysis.
