# Supplementary material for: Olfactory Receptor OR2K2 Expression in Human Choroid Plexus as a Potential Marker in Early Sporadic Alzheimer’s Disease
Source: Genes (Basel). 2024 Mar 21;15(3):385. doi: 10.3390/genes15030385 (PMC10970182; doi:10.3390/genes15030385)
Supplement: Supplementary file 1 [file genes-15-00385-s001.zip › genes-2891338-supplementary.pdf]

Table S1 - List of primer sequences used for Selfie-digital PCR analysis.

Gene		Primer sequences (5' to 3')	Target exon
Taste receptors, type 1			
TAS1R1	Fw	TGCCAGAGAACTACAACGAGG	6/5
	Rv	GTGGTGAAGAAGGCGATCCA	
	Fw	ATGTGTATGCCACGCTGAGA	
	Rv	GCACCGTAGGGGAATAGTGG	
TAS1R2	Fw	CTGTAACCCCAACTACCGCA	6
	Rv	CCCATGTAGGCGAAGCTGAA	
TAS1R3	Fw	CAGGGCTAAATCACCACCAGA	3
	Rv	CTGAGGCGTTGCACTGAAGA	
Taste receptors, type 2			
TAS2R5	Fw	TTACGGAAGGACGAGGCCAA	1
	Rv	AGGTCAGATCCCCTGGTAGTG	
TAS2R14	Fw	GACTGGGTCAAGGGAAGAAAGA	1
	Rv	ACCAAACCAGGCTAATTCGAGA	
Olfactory receptors			
OR1L8	Fw	ACCGTGGTGACGCTCTTTTA	1
	Rv	TTGACAGCGTAGGTGGATGG	
OR2H2	Fw	GGCACTCCTTCACTTAACCC	1
	Rv	TGTGTGAGCCCCATTTCCTT	
OR2K2	Fw	TGGTTCTCTTCGTCTTCAGCC	1
	Rv	CATGGGGGTTTTAAGGCGTG	
OR7A5	Fw	CATGCCTGTCTCGTTTCCCT	1
	Rv	TGTGTATCATTTCCTGGTTCCATTT	
OR7A17	Fw	AACCAGAGAATGACACAGGGATTT	1
	Rv	CATGGACAGAAACAGCCCAAAG	
OR13A1	Fw	CTGCGCTTGGATTTCTGTGG	4
	Rv	CACCGTTGACGTAGGTGGAG	

Fw, forward primer; Rv, reverse primer.

(a)

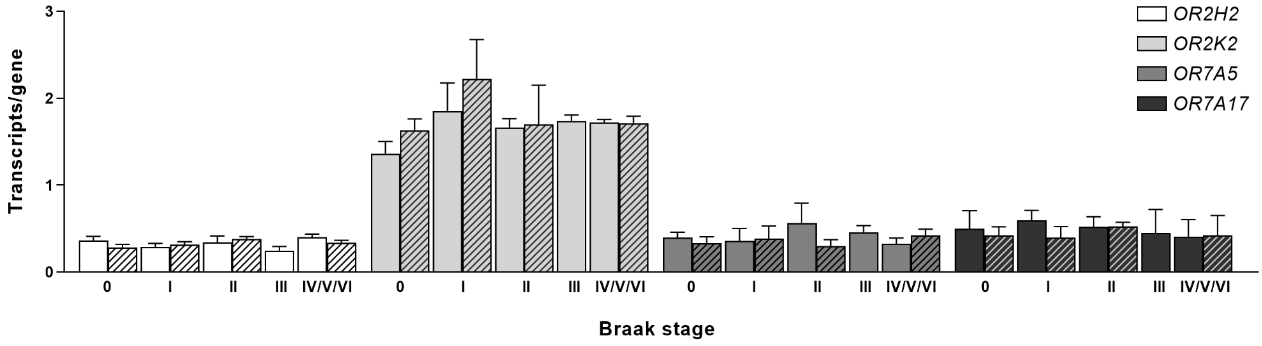

(b)

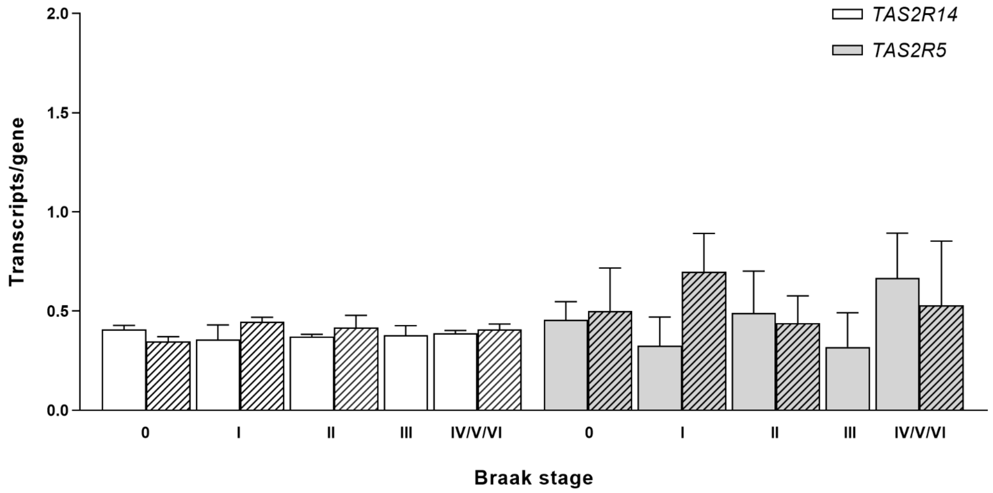

Figure S1. Comparison of chemoreceptors expression between males and females. Absolute quantification of *OR* **(A)** and *TAS2R* **(B)** genes transcription was conducted using selfie-dPCR. Solid bars denote data from males, while dashed bars represent females. Males and females were compared across each Braak stage using one-way ANOVA followed by Sidak multiple comparison test.

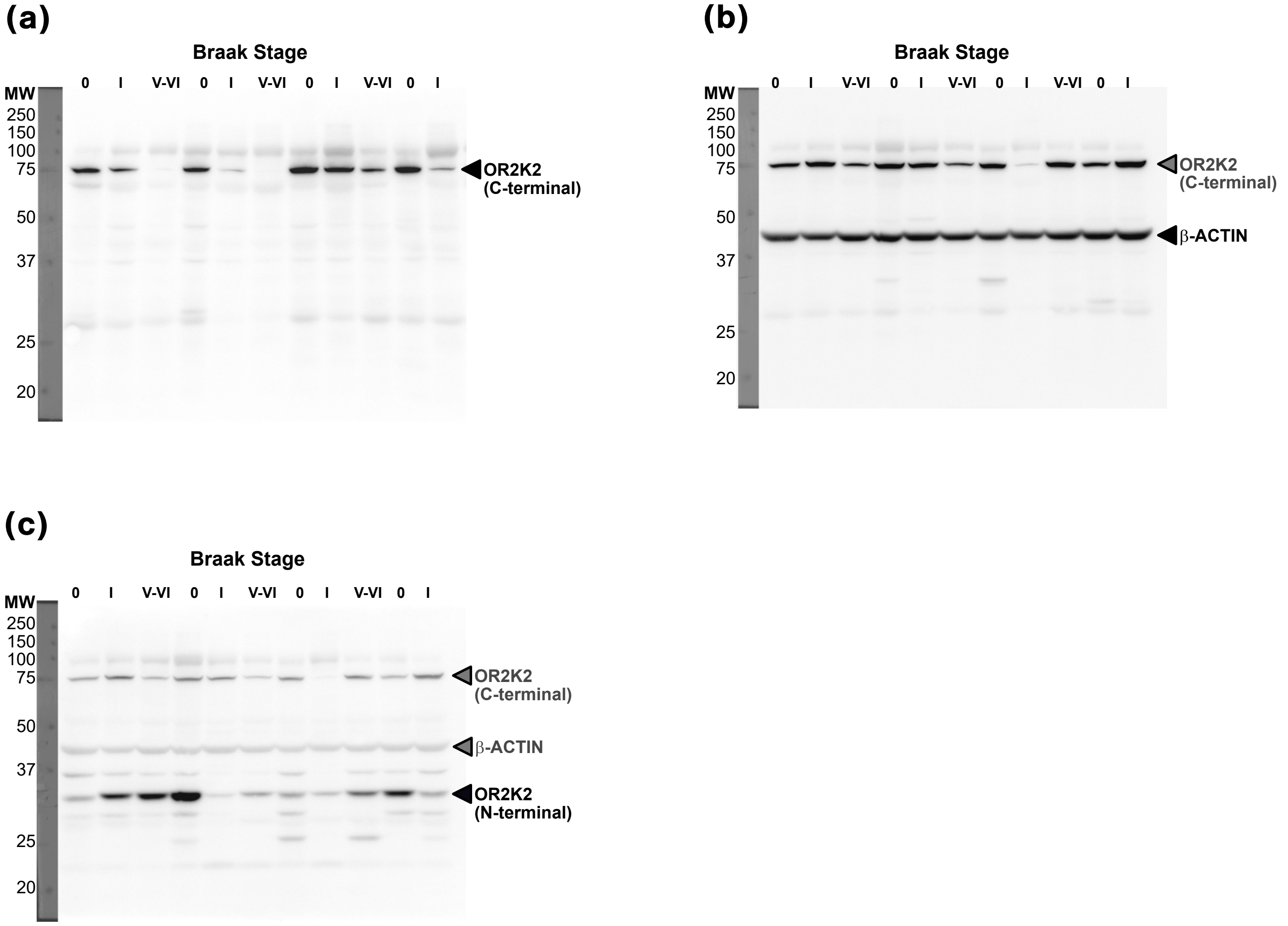

Figure S2. Quantification of OR2K2 in the Choroid Plexus Across Braak Stages. Western blot analysis was employed to assess the relative protein levels of OR2K2 in the choroid plexus at various Braak stages. Two distinct antibodies were utilized, with ß-Actin serving as protein loading control. **(A)** Full immunoblot of OR2K2 using the C-terminal antibody. **(B)** Full immunoblot of ß-Actin. **(C)** Full immunoblot of OR2K2 using the N-terminal antibody, with this membrane re-probed from the immunoblot in panel B. Solid black arrows and accompanying names indicate the protein being blotted, while solid grey arrows and names denote proteins from previous blots. Molecular weights (MW) are represented by a ladder (kDa) for reference.
